# Does case misclassification threaten the validity of studies investigating the relationship between neck manipulation and vertebral artery dissection stroke? Yes

**DOI:** 10.1186/s12998-016-0123-x

**Published:** 2016-11-05

**Authors:** Jessica K. Paulus, David E. Thaler

**Affiliations:** 1 Predictive Analytics and Comparative Effectiveness (PACE) Center, Institute for Clinical Research and Health Policy Studies (ICRHPS), Tufts Medical Center/Tufts University School of Medicine, Boston, MA USA; 2 Department of Neurology, Tufts Medical Center/Tufts University School of Medicine, 800 Washington St, Box 314, Boston, MA 02111 USA

**Keywords:** Case misclassification, Administrative data, Claims, Arterial dissection, Chiropractic, Spinal manipulation therapy

## Abstract

**Background:**

For patients and health care providers who are considering spinal manipulative therapy of the neck, it is crucial to establish if it is a trigger for cervical artery dissection and/or stroke, and if it is, the magnitude of the risk.

**Discussion:**

We discuss the biological plausibility of how neck manipulation could cause cervical artery dissection. We also discuss how case misclassification threatens the validity of influential published studies that have investigated the relationship between neck manipulation and dissection. Our position is supported by the fact that the largest epidemiologic studies of neck manipulation safety with respect to neurological outcomes have relied on International Classification of Diseases-9 codes for case identification. However, the application of these codes in prior studies failed to identify dissections (rather than strokes in general) and so conclusions from those studies are invalid.

**Conclusion:**

There are several methodological challenges to understanding the association between neck manipulation and vertebral artery dissection. Addressing these issues is critical because even a modest association between neck manipulation and cervical artery dissection could translate into a significant number of avoidable dissections given the widespread use of neck manipulation by providers from various backgrounds. We believe that valid case classification, accurate measurement of manipulative procedures, and addressing reverse causation bias should be top priorities for future research.

## Background

### Blinded by the SUN

Imagine that Australian researchers believe that there is a higher risk of developing melanoma on their continent because of the increased amount of sun exposure in their environment. Small, observational studies have suggested a link. Ultraviolet exposure in laboratory environments is known to produce mutagenic and cytotoxic DNA lesions. They might set up an observational study, the Sun down Under and Neoplasm (SUN) Study, comparing the incidence of disease in white-skinned Australians versus a similar ethnic group, say the Scottish in northern Britain, where sun exposure is lower. Because melanoma is a relatively rare cancer, they need a large dataset to gather enough cases for the analysis. They discover that the incidence of disease is the same in the two countries. The inescapable conclusion, they argue, is that given the large difference in sun exposure and the same risk of disease, sun exposure is excluded as a risk factor. However, what if it turned out that the cases identified as having “the disease” were actually anyone with a diagnostic code for cancer-not just melanoma. Even given a strong, causal relation between sun exposure and melanoma, this hypothetical investigation was doomed. Other cancers may be more common in Scotland (e.g. lung cancer due to more smokers there than in Australia) and that could obscure a truly higher rate of melanoma in Australia. Malignant skin cancers have a much lower incidence than, say, lung cancer (by roughly an order of magnitude) and so detecting a sun-related signal affecting a small portion of the study population would be almost impossible. Given this flaw in the case identification scheme, it would not take long for most to decide that the SUN Study is largely uninformative with regard to the risk of sun exposure on skin cancers. To those who are aware of how the SUN Study was conducted, it would be jarring to read, years later, repeated assertions in the cancer literature that there were “identical rates of melanoma in Scotland and Australia”.

### Discussion

The fictional story above is directly analogous to the debate regarding cervical spinal manipulative therapy (SMT) and cervical artery dissection (CAD). The possibility that SMT causes CAD has concerned neurologists for decades, with arguments referencing the size of the association, temporality, biological plausibility and consistency of observation in support of causal inferences [1]. Four epidemiological studies [1–4], have indicated associations with adverse neurological events, including arterial dissection and stroke. Yet three other epidemiologic studies-with the largest sample sizes-indicate no increased risk associated with SMT [5–7]. While a first instinct might be to trust the larger studies, we argue that a closer look is needed, and that case misclassification may partly explain these discrepant findings, rendering some of them altogether uninformative to the dissection debate.

One of the biggest challenges in accurately classifying dissection cases stems from the rarity of arterial dissection itself. There is an estimated annual incidence of only 2.5–3 cases of carotid artery dissection per 100,000 and 1–1.5 cases of vertebral artery dissection per 100,000 [8]. Because of this low incidence rate, large populations must be screened to collect enough cases to furnish adequate statistical power for research investigation. Yet larger study populations are most practically assembled through the use of secondary data, or data collected primarily for purposes other than research (ie claims data). As a general principle, with larger quantities of data come challenges in assuring their validity and integrity [9, 10], while biases due to measurement error are independent of the volume of data [11]. Routinely collected electronic data offer an efficient, low-cost approach for identifying large numbers of cases but there is a greater risk of error as compared to the more taxing strategy of verifying disease status for each individual. This tradeoff between the numbers of cases and the case classification strategy is readily evident in the published literature on the safety of SMT.

Arterial dissection, rather than stroke in general, is the relevant disease outcome of interest with respect to evaluating potential SMT safety. Stroke is an important cause of acquired disability and death in the United States and elsewhere, but is not a disease-it is a consequence of many other conditions. Common stroke mechanisms include small artery (lacunar) disease, large vessel atherosclerosis, and cardiogenic embolism. Cervical artery dissection, the separation of the layers of the arterial wall supplying blood to the head and brain, is a relatively uncommon cause of stroke in the general population (~2 %), but accounts for up to 25 % of strokes in young and middle-aged patients.

Traumatic CAD occurs because internal soft tissues move when there is movement of the neck. The carotid and vertebral arteries are partially anchored and partially mobile so when the neck moves, the arteries do too. There is the capacity in the cervical arteries to stretch and so to be structurally unaffected by normal movement. However, if the movement is more than “normal” then that capacity can be overwhelmed and the vessel can become structurally affected. How much movement it would take for an individual artery to become injured is unpredictable. People differ with regard to many of the relevant parameters that would affect this risk-hydration status, arterial stiffness, length of the neck, length of the arteries, redundancy of arteries (eg loops), and the size and location of adjacent anchoring structures such as cervical muscles, styloid processes, osteophytes and vertebrae. Stretching promotes injury to the intimal layer of the arterial wall, which can become separated from the medial layer. Blood flowing in the lumen then dissects the intima from the media separating them further. Intramural hematoma is the pathological finding in dissection. Experimental preparations with small numbers of cadavers [12], or in vitro preparations of vertebral artery segments measured with strain gauges, cannot entirely account for the myriad internal variations of anatomy that might predispose to dissection.

What sort of “trauma” is necessary to produce a stretch injury? It is well recognized that major trauma, such as a motor vehicle accident, is sufficient. Head and neck movement in such circumstances can exceed the anatomical constraints of a vessel and lead to dissection. Direct trauma from a cricket ball and a lateral rotational movement led to the injury and tragic death of Phillip Hughes, a 25-year-old Australian batsman who suffered a vertebral artery dissection as the result of a “bouncer” in 2014. However, much milder traumas have also been described as mechanical triggers of dissection including abnormal positions of the neck during prolonged telephone calls, yoga movements, vigorous Valsalva during lifting or vaginal deliveries, and even hyperextension at the beauty parlor sink [13, 14]. Given these known precipitants, it is biologically plausible that mechanical nature of cervical manipulation is another potential trigger of dissection. Spinal manipulation or adjustment techniques are a cornerstone of chiropractic care in the United States and Canada [15–17], with almost one quarter of the clinical training contact hours in chiropractic curricula devoted to this treatment [18]. Although less commonly used by physiotherapists [19], curricula have been proposed to increase the use of SMT [20]. During cervical SMT, patients’ heads are typically put into a position that includes some combination of extension, lateral rotation, lateral flexion, and traction [21]. The position may be beyond the range that is possible with active movement (by the patient themselves), but still within the range of normal passive joint flexibility. The manipulation then takes place with a single high-velocity, low amplitude thrust that sends the joints slightly beyond the range of normal passive movement. This may lead to the release of gas from the joint space producing an audible “pop” or cavitation. It requires no violation of physiological or anatomical principles to conclude that during that moment of increased stretch, just beyond the limit of passive movement, in a person who is anatomically and/or physiologically susceptible, the integrity of the arterial intima can become compromised and a dissection can occur.

Neurological symptoms of dissection occur in two ways: 1) local compression by the hematoma of adjacent structures (eg Horner syndrome from disruption of the sympathetic fibers around the carotid artery) or 2) arterial stenosis or occlusion, which can produce cerebral ischemia directly or after embolization of a part of the thrombus. Many patients with dissection will have only neck or head pain (without neurological symptoms) and may go undiagnosed. Some studies of patients with confirmed CAD indicate that only 50 % experience cerebral or retinal ischemia [13].

The accurate diagnosis of arterial dissection requires a high degree of clinical suspicion, neurological examination, and neuroimaging. The characteristic history includes a new kind of unilateral head or neck pain, not exacerbated by movement. Symptoms and/or signs of focal cerebral ischemia in association with such pain, especially in a demographic less prone to conventional stroke mechanisms (younger, fewer vascular risk factors) are particularly suggestive. Neuroimaging is often specialized and the findings are dynamic, making the timing of the diagnostic study almost as important as the selection of which study to perform [13]. Vascular imaging includes not just visualization of the lumen, as in Computed Tomography (CT), Magnetic Resonance Imaging (MRI), or conventional contrast angiography, but also visualization of the arterial wall such as with T1-weighted MRI sequences with fat suppression of the cervical soft tissues.

The largest epidemiologic studies of SMT safety with respect to neurological outcomes have relied on International Classification of Diseases-9 codes for case identification [5–7]. However, using ICD codes from hospital discharges to accurately identify strokes, and to determine stroke etiology, is unreliable [22–27]. There is a lack of consensus among stroke studies about which codes should be used to identify outcomes [25], heterogeneity in the performance of codes across populations and stroke subtypes [27], and evidence that case-finding algorithms should be tailored for each stroke subtype of interest [28]. Using ICD-9 codes to identify arterial dissections is particularly problematic. Although there are diagnostic codes specific to dissections of the vertebral and carotid arteries, a clinical encounter with a dissection patient may be reasonably coded as neck pain, headache, occluded cerebral artery, stroke, transient ischemic attack (TIA) or another non-specific diagnosis that would severely challenge their identification as a dissection from administrative codes alone.

The ICD-9 codes used in the largest epidemiological studies [5–7] were related to vertebrobasilar stroke in general-with its many different etiologies, instead of codes specific for dissection. The codes used were specific for a neurovascular location (posterior circulation), rather than for a vascular diagnosis (dissection). As not all posterior circulation strokes are caused by dissection, and not all dissection-related strokes are in the posterior circulation, this strategy would lead to extreme case misclassification because most cases would not be due to dissection. The most recent case-control study to use this strategy [7] compounded the earlier error by excluding subjects who were assigned with the dissection-specific codes [29] . The earlier studies [5, 6] took place in Ontario, at a time when the ICD-9 codes specific for dissection (443.XX) were not in use. Patients in Canada, clinically diagnosed with dissection, would have been coded with the posterior circulation codes that were used for their case identification. The more recent study [7] was done in the United States where the dissection-specific codes are in widespread use. Patients with clinical CAD diagnoses would have been most accurately coded with a 443.XX code and not with the anatomically-based posterior circulation codes used in Canada. Because the dissection codes were not added to the case identification strategy (their aim was to replicate the earlier study and so they adhered closely to the published protocol) patients with CAD were systematically excluded. It follows that the positive predictive value of true CAD patients in the US study is likely to be even lower than that which was observed using the Canadian case identification strategy.

Since stroke from other mechanisms are unlikely to be affected by SMT, this misclassification would lead to an underestimation of any true association between SMT and dissection. That these codes likely identified stroke patients in general, rather than patients with arterial dissection, is supported by higher observed prevalences of cardiovascular risk factors such as hypertension, diabetes and ischemic heart disease in the cases versus controls. In one study, cases were 1.5 to 2 times more likely to have hypertension, ischemic heart disease, endocrine disease, or hypercholesterolemia than controls [7]. This is the opposite of what is usually seen in well-identified dissection patients, who tend to be young and relatively healthy.

The accuracy of diagnostic codes is often quantified using the positive predictive value (PPV)-or the proportion of code-identified cases that are true cases according to a gold standard. For assessing stroke trends over time, a PPV ≥85 % has been proposed as adequate [30]. Assuming that the misclassification is random with respect to risk factor status (ie the codes are not more or less accurate for participants receiving SMT versus not), similarly high PPVs (≥90 %) are anticipated to introduce minimal bias for studies comparing the efficacy or safety of one treatment versus another [11]. We reviewed nearly 3700 “cases” identified by using the same ICD-9 code defined strategy in the Veterans Health Administration electronic medical record database. We found that only about 400 had confirmed CAD following neurologists’ reviews of medical records and neuroimaging data-a PPV of only 11 % [31]. Assuming this misclassification was random with respect to SMT status, the prior case-control studies would have a significant bias towards the null. Our sensitivity analysis suggested that if we applied a PPV of 11 % to “correct” the results of one of the prior studies [5], then the reported null odds ratio of 1.1 could be as high as 2.2. Our study publication acknowledges the possible limits of generalizability from the VA population to the one in Ontario but also points out that the limitations may have led to an underestimation of the CAD/SMT association [31].

Several case-control studies have used more reliable case definitions that include radiographically-confirmed dissection [2–4, 32]. While most studies with imaging or medical record confirmed cases are limited by small sample sizes, it is striking that all four report that SMT is associated with a higher risk of CAD. The Cervical Artery Dissection and Ischemic Stroke Patients (CADISP) consortium case-control study included nearly 1 thousand radiographically-confirmed CAD patients and also found significantly elevated risk of CAD associated with SMT [4]. However, CADISP did not review SMT records to confirm if, and what type of manipulations occurred. Therefore, inaccurate assignment of exposure to the putative trigger (SMT) is possible, and recall bias may have contributed to their data given the study design. A recent meta-analysis pooling all published studies found a nearly two fold higher risk of CAD associated with SMT [33]. But when limited to the 4 studies with imaging confirmation of cervical arterial dissection as the endpoint, SMT was associated with a significant 4-fold increase in risk [34].

Another argument to explain the epidemiological association between SMT and CAD is protopathic bias, or reverse causation. Because a dissection can produce headache or neck pain, patients who have already had a dissection may seek out care with chiropractors and others. The CAD diagnosis is then subsequently made. This, indeed, is a plausible explanation for some patients. It would not explain, though, why patients who receive SMT for reasons other than neck/head pain, say for lumbar complaints or for “maintenance,” would end up with a cervical dissection. Such a sequence of events has been observed by neurologists, including one of us. A case series found that 9 % of patients with SMT-associated dissection did not have head or neck pain as the indication for their treatment [35]. Also unexplained by the reverse causation explanation is why the SMT-CAD association would be observed in young (<45 years) but not in older (>45 years) patients [5], as it is not clear why reverse causation bias would differ by age. However, it would be expected that the ICD-9-based case identification strategy would have a greater PPV among young stroke patients (who have a much higher prevalence of dissection) than in older populations. Such a differential PPV by age would allow an association to be detectable (though still underestimated) in the younger group but not in the older one.

Finally, the reverse causation argument is problematic for another reason. It is well recognized in chiropractic teaching that cervical manipulation should not be administered to patients with a pre-existing dissection [36, 37]. The concern is for exacerbating the dissection or for disturbing the mural/luminal thrombus such that a portion of it could be liberated and embolize into the brain (or retina, in the case of a carotid dissection). If patients are presenting to chiropractors because of arterial dissections, then it is crucial that such patients be identified before being manipulated. Otherwise some proportion of patients with a known absolute contraindication will be subjected to a hazardous treatment. Such patients are potentially identifiable with a careful history and neurological examination and targeted investigations. If one argues that neck and head pain from CAD is indistinguishable from pain caused by more mundane musculoskeletal conditions, then this is a tacit acknowledgment of an intolerable situation, that some patients will be inevitably harmed by the procedure.

Establishing whether or not SMT is a risk factor for CAD, and if so, the magnitude of the risk, is crucial for patients and health care providers considering SMT. Given the prevalence of SMT exposure (~8 % of adults and ~3 % of children annually) [38], even a modest increase in risk of dissection associated with SMT [33] could translate to thousands of avoidable dissections and strokes worldwide each year. So, on a population level, even a modest association between SMT and CAD could translate into a meaningful impact on public health. While there are several methodological challenges to this question [33, 39]-including accurate measurement of SMT procedures, and reverse causation-valid case classification should be a top priority. There are several ways forward to address the data quantity-quality tradeoff. First, an emphasis should be placed on accurate case identification using detailed clinical and radiographic criteria. This may require collaboration across research sites in order to obtain sufficient numbers for analysis. Second, if studies must rely on case-identification strategies that use only administrative datasets, and without individual case confirmation, then they must evaluate the validity of the strategy, and adjust their results for misclassification bias [10]. Lastly, while not the focus of this article, defining and measuring the relevant exposure - cervical manipulation (perhaps even a subtype of manipulation) - should be as rigorous as defining the dissections themselves so that the most accurate picture of the relationship can emerge.

It turns out that more time in the sun really can cause melanoma. The reassurance from the (fictional) SUN Study would have diverted attention away from the truth. Better case definition would have led in the right direction. The American Chiropractic Association prioritizes one of the studies [6] relying on ICD-9 code case identification as the “most credible research study to date,” [40] and suggests incorporating these findings in the provider-patient discussion of SMT safety. We strongly urge a critical review of the case definitions that have been used in this field so that our non-fictional studies don’t become similarly distracting. If SMT causes CAD, we need to know.

## Conclusion

On a population level, even a modest increase in dissection risk triggered by neck manipulation could translate into a meaningful impact on public health given the widespread use of SMT by diverse health care providers. There is biological plausibility in why neck manipulation could cause vertebral artery dissection but importantly case misclassification threatens the validity of some of the previous studies investigating the relationship between neck manipulation and vertebral artery dissection. Valid case classification, accurate measurement of manipulative procedures, and controlling for reverse causation should be top priorities for future research.

## Abbreviations

CAD: Cervical artery dissection
CADISP: Cervical Artery Dissection and Ischemic Stroke Patients
CT: Computerized tomography
MRI: Magnetic resonance imaging
SMT: Spinal manipulative therapy
TIA: Transient ischemia attack
